# Supplementary material for: Identification of DREB Family Genes in Banana and Their Function under Drought and Cold Stress
Source: Plants (Basel). 2024 Jul 31;13(15):2119. doi: 10.3390/plants13152119 (PMC11314547; doi:10.3390/plants13152119)
Supplement: Supplementary file 1 [file plants-13-02119-s001.zip › Table S1.pdf]

Function	Software	Website
Genome Data of MaDREB	Banana Genome Database	https://banana-genome-hub.southgreen.fr/
Identification of MaDREB Gene Family Members	HMMER	http://www.hmmer.org/
	PFAM database	http://pfam.xfam.org/
	TAIR	http://www.arabidopsis.org/
	NCBI database	https://www.ncbi.nlm.nih.gov/Structure/cdd/wrpsb.cgi
Phylogenetic Analyse	MEGAX software	
Conserved Motifs	MEME	http://meme-suite.org/tools/meme
	NCBI CDD searching	https://www.ncbi.nlm.nih.gov/Structure/cdd/wrpsb.cgi
Intron-exon Organization	TBtools	/
Cis-Acting Elements Analysis	PlantCARE database	http://bioinformatics.psb.ugent.be/webtools/plantcare/html/
Protein Collinearity Network Analysis	orthovenn2 tools	https://orthovenn2.bioinfotoolkits.net/home
	AraNetV2	http://www.inetbio.org/aranet/
	String software	https://string-db.org/
	Cytoscape software	https://cytoscape.org/
Gene Collinearity Analysis	MCScanX software	/
